# Intraoperative Analgesia with Magnesium Sulfate versus Remifentanil Guided by Plethysmographic Stress Index in Post-Bariatric Dermolipectomy: A Randomized Study

**DOI:** 10.1155/2022/2642488

**Published:** 2022-10-26

**Authors:** S. E. Silva Filho, S. Dainez, M. A. M. C. Gonzalez, F. Angelis, J. E. Vieira, C. S. Sandes

**Affiliations:** ^1^Department of Anesthesiology, Hospital da Sociedade Portuguesa de Beneficência de Santos, Santos, SP, Brazil; ^2^Department of Anesthesiology, Universidade de Sao Paulo, Santos, SP, Brazil; ^3^Hospital Santos Dumont, Sao Jose Dos Campos, SP, Brazil

## Abstract

**Background:**

Magnesium sulfate reduces pain scores and analgesic consumption. Its use as an analgesic resource in opioid-free or opioid-sparing techniques have also been tested. The evaluation of the antinociceptive potency of drugs and doses indirectly, through hemodynamic oscillations has been questioned. A relatively new algorithm called the plethysmographic stress index has been considered sensitive and relatively specific as a parameter for assessing the need for intraoperative analgesia.

**Objectives:**

The aim of this trial was to assess the intraoperative analgesic capacity of magnesium sulfate compared to remifentanil. The secondary objectives were propofol consumption and its latency, the consumption of opioids, ephedrine, and cisatracurium. *Patients and Methods*. Forty patients scheduled for post-bariatric dermolipectomy were randomly assigned to two groups to receive total intravenous anesthesia with target-controlled hypnosis induced with propofol. Analgesia was obtained in the remifentanil group with remifentanil at an initial dose of 0.2 *μ*g·kg^−1^·min^−1^ and in the magnesium sulfate group with magnesium sulfate 40 mg·kg^−1^ + 10 mg·kg^−1^·h^−1^.

**Results:**

There was no statistical hemodynamic difference between the groups before and after orotracheal intubation (*p* = 0.062) and before and after the surgical incision (*p* = 0.656). There was also no statistical difference in the variation of mean arterial pressure before and after intubation (*p* = 0.656) and before and after the surgical incision (*p* = 0.911). There was similar consumption of cisatracurium, ephedrine, and postoperative opioids between the groups. Some patients in the magnesium sulfate group needed more intraoperative fentanyl and propofol, although the latency of propofol was similar in both the groups.

**Conclusion:**

We conclude that using magnesium sulfate in intravenous general anesthesia for post-bariatric dermolipectomy is related to a significant reduction in opioid consumption without compromising hemodynamic stability. Overall, PSI monitoring was helpful in driving the analgesic strategy. The use of magnesium sulfate proved to be an important adjunct in the scenario presented, allowing the use of opioids to be avoided in certain cases. We found no statistical differences in the consumption of neuromuscular blocker and vasoconstrictor. Substituting opioids for magnesium sulfate leads to an increase in propofol consumption in the scenario presented. Studies with a larger sample are needed to corroborate the results presented and evaluate other potential advantages in reducing opioid consumption.

## 1. Introduction

Magnesium sulfate has shown its usefulness as an adjuvant in the treatment of several medical conditions, like cardiac arrhythmias [1], eclampsia [2], pulmonary hypertension [3], pheochromocytoma [4], asthma [5, 6], and in fetal neuroprotection [7]. In recent years, it has been explored as an adjuvant in anesthesia owing to several effects (analgesic, opioid-sparing intra and postoperative, hypnotic-sparing, antihyperalgesic, and muscle relaxation potentiating effect among others) [8, 9]. It seems to cause most of these effects, acting as an antagonist at calcium channels and as a blocker at N-methyl-D-Aspartate (NMDA) receptors [10–16]. Among the advantages of its use are the reduction of opioid consumption and, consequently, its side effects (for example, nausea and vomiting, respiratory depression, gastrointestinal impairment, postoperative hyperalgesia, addiction, and pruritus) [17–20]. Magnesium sulfate could also provide greater hemodynamic stability when compared to remifentanil during post-bariatric dermolipectomy [21].

The evaluation of the intraoperative nociception and antinociceptive effect of the drugs and doses is usually focused on hemodynamic responses (arterial blood pressure and heart rate), tearing, movements or, when available, the difference between response entropy and state entropy. All of them are indirect parameters, frequently affected by other phenomena. Thus, as surrogate information, they lack specificity [22]. A relatively new algorithm called the plethysmographic stress index (PSI) has been considered sensitive and relatively specific as a parameter for assessing the need for intraoperative analgesia. It is based on normalized pulse photoplethysmographic amplitude and heartbeat intervals. The range of the PSI scale goes from 0 (low stress) to 100 (high stress), and the target range is between 20 and 50 [22–26].

The analgesic capacity of magnesium sulfate has not yet been tested using the PSI algorithm. The primary objective of this trial was to assess the intraoperative analgesic capacity of magnesium sulfate compared to remifentanil with assessment using this algorithm. For this, variations in heart rate (HR) and mean arterial pressure (MAP) caused by intubation and surgical incision and fentanyl (used as a rescue analgesic) consumption were compared. The secondary objectives were to compare postoperative pain scores, postoperative morphine, cisatracurium, ephedrine, and intraoperative propofol consumption, in addition to latency to hypnosis.

## 2. Methods

The project of this study was approved by the Research Ethics Committee of the University of São Paulo, SP, Brazil (Certificate of Presentation for Ethical Appreciation—12614719.1.0000.0068, approved according to opinion no.: 3,731,805) and the free and informed consent form was obtained from all participants in this trial. The approved project was registered with clinicaltrials.gov (NCT04005599) before the start of participant recruitment. This prospective, controlled, randomized study was blinded to the participants, the medical staff who performed the anesthesia, who collected the data, and who analyzed it. The elaborate PICO question (population, intervention, comparison, and outcome) was “In patients undergoing post-bariatric dermolipectomy, the administration of magnesium sulfate as the intraoperative analgesic agent can block nociceptive stimuli, compared to remifentanil, maintaining surgical stress rates between 20 and 50?” This manuscript is based on the CONSORT guideline.

### 2.1. Study Population

Patients aged between 18 and 60 years, classified by the American Society of Anesthesiologists (ASA) status I or II and body mass index (BMI) < 35 kg·m^−2^, scheduled for post-bariatric dermolipectomy, fit, and who agreed to sign the informed consent form were invited to participate. The exclusion criteria include the following: history of allergy to any component of the study protocol, refusal to participate in the study or sign the informed consent form, neuromuscular disorders, heart block other than first-degree atrioventricular block, use of illicit drugs, history of chronic pain, psychiatric disorders that made it difficult to assess symptoms, use of calcium channel blockers, and renal failure. Recruitment and data collection were performed at Hospital Santos Dumont in São José dos Campos, SP, Brazil, between July 3, 2019, and January 6, 2022.

### 2.2. Sample Size

**Figure 1:**
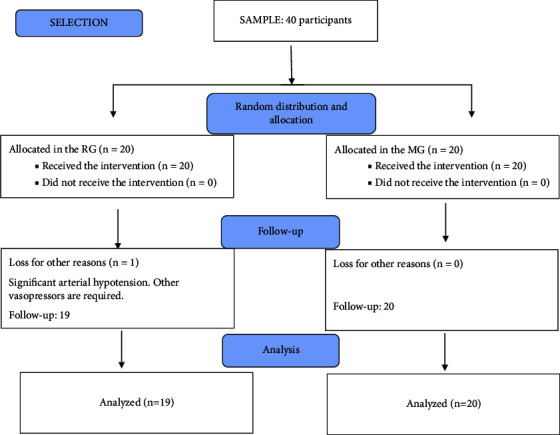
Consort flowchart. RG, remifentanil group; MG, magnesium sulfate group.

The sample size calculation was based on a previous study carried out by the authors, with a similar protocol but without PSI monitoring [21]. Hemodynamic stability was found in the groups that received total intravenous anesthesia associated with remifentanil or magnesium sulfate, but at the expense of higher consumption of propofol in the group magnesium sulfate (123.33 *μ*g·kg^−1^·min^−1^ ± 15.68 *μ*g·kg^−1^·min^−1^ versus 90.07 *μ*g·kg^−1^·min^−1^ ± 19.94 *μ*g·kg^−1^·min^−1^; *p* < 0.001). Using Pocock's formula [27] (Figure 1), a 95% confidence level and 90% statistical power and a sample of 8 were calculated. We decided to view this trial as a pilot to evaluate the hemodynamic behavior of patients with PSI-controlled analgesia, and we increased the sample to 20 participants in each group. Since the two techniques compared were already being used in our service, there would be no ethical conflict or economic implications.

### 2.3. Allocation

The sample was randomly distributed through an electronic lottery on the website https://www.random.org/, which uses an atmospheric noise algorithm and was divided into two groups, called remifentanil group (RG) and magnesium sulfate group (MG). The execution of the draw and its result were the responsibility of a member of the anesthesiology team who prepared opaque envelopes numbered according to the result of the covertly draw for all other team members and the patients. Inside each envelope, there would be a card with the group's name and the conduct corresponding to the envelope number. The envelope would only be opened in the operating room by a professional unrelated to other stages of the study, who would only prepare the covered solution, following the instructions on the internal card. Participants and the anesthesiologists (provider, responsible for collection or for data analysis) were unaware of the group and intervention of each participant.

### 2.4. Anesthetic Technique

All patients in the study were monitored with continuous electrocardiography, noninvasive blood pressure, pulse oximetry, capnography, and neuromuscular relaxation through a sequence of four stimuli (train of four-TOF-sensor NMT Mechano Sensor Ref 888414, GE) on the ulnar nerve/adductor pollicis muscle, awareness level monitoring through entropy (PROCARE B 20 Monitor, E-PSMP module, EasyFit Entropy Sensor, GE), and nociception through PSI (Datex-Ohmeda S/5 Anesthesia Monitor, S/5 iCentral Network Workstation and S/5 iCollect data acquisition software, GE Healthcare Finland Oy, Helsinki, Finland).

After recording the baseline monitoring data, peripheral venous access was performed with a 20-or 18-gauge catheter, and the first blood sample was collected, following a 250 ml saline solution infusion covertly to finish in 15 minutes (in the RG group, without magnesium sulfate; in the MG group, with 40 mg·kg^−1^ of magnesium sulfate, information found inside the envelope, in a hidden way for the patient and the team). Concomitantly, premedication with 2 g dipyrone, 2 *μ*g·kg^−1^ clonidine, 4 mg dexamethasone, 2 g cefazolin, 4 mg ondansetron, and 100 mg ketoprofen was administered. After the end of the saline solution infusion, the infusion was started using a syringe pump with a solution previously prepared in 60 ml syringes at a speed of 0.2 ml·kg^−1^·h^−1^. This rate corresponded, due to the prepared dilution, to magnesium sulfate 10 mg·kg^−1^·h^−1^, in MG, or remifentanil 0.2 *μ*g·kg^−1^·min^−1^, in RG. The solutions were prepared like the following:

- RG: 250 ml saline solution for infusion over 15 minutes. Plus: 60 ml syringe with 33 ml of saline solution + 2 mg remifentanil.
- MG: 250 ml of saline solution + magnesium sulfate 40 mg·kg^−1^. Plus: 60 ml syringe with 16.5 ml of saline solution + magnesium sulfate 10% 16.5 ml.

After recording the baseline monitoring data, 1.5 mg·kg^−1^ of lidocaine was administered, and a target-controlled infusion of propofol was initiated for a mean initial target concentration of 4 *μ*g·ml^−1^ (Marsh effect model). When entropy reached values between 40 and 60, the infused dose of propofol and the hypnosis latency were recorded, the TOF was calibrated, and cisatracurium was administered at a dose of 0.15 mg·kg^−1^. When monitoring of neuromuscular function showed zero counts, entropy and PSI values were recorded, followed by orotracheal intubation. The time between cisatracurium administration and TOF 0 was noted. After orotracheal intubation, propofol infusion was guided by hypnosis monitoring. The infusion of the covert solution was controlled for PSI values between 20 and 50. Elevations of PSI above 50 with entropy between 40 and 60 were treated with increased infusion of the covert solution. If the infusion rate reached 0.4 ml·kg^−1^·h^−1^ (double the initial rate) and the PSI remained above 50, 1 *μ*g·kg^−1^ of fentanyl was administered until control. Reduction of the PSI below 20 was treated with reduction of the covert solution. Hypotension associated with adequate hypnosis and a PSI between 20 and 50 was treated with ephedrine in doses of 5 mg and bradycardia with atropine 0.5 mg. A booster dose of cisatracurium (0.03 mg·kg^−1^) was administered in the following situations: TOF ≥ 1, surgeon's request or patient's respiratory effort detected. At the end of the surgery, the infusions of propofol and the covert solution were interrupted. When TOF monitoring showed two responses, the patient received neostigmine 0.03 mg·kg^−1^ associated with atropine 0.15 mg·kg^−1^. The following were recorded: total cisatracurium administered in the procedure, the time between the start of propofol infusion and anesthetic hypnosis, total consumption of propofol, total consumption of fentanyl, total consumption of ephedrine, and pain on awakening, after 6 h and in the three days following the day of surgery (at rest and movement, morning and afternoon). Pain assessment in this trial was performed using the VNS, whose principle was previously explained to patients at the time of recruitment and reinforced at each pain assessment. Pain on awakening greater than 3 (verbal numerical scale-VNS-from 0 to 10; 0 no pain and 10 the most intense pain imaginable) was treated with dipyrone 2 g, and if the pain persisted, morphine 2 mg every 20 min until pain resolution, or, after the third dose, patient-controlled analgesia (PCA) with morphine will be started. HR and MAP values were also measured before and after orotracheal intubation and before and after the surgical incision.

Anthropometric data (age, weight, height, and BMI) and ASA status classification were collected for comparison between the groups.

The data collected were organized in a spreadsheet (MS-Excel, version MS-Office 2013). All statistical tests and graphics were performed in SPSS V26 and R 3.6.0 software. All results with a descriptive level lower than 5% (*p* < 0.05) were considered significant.

## 3. Results

Forty patients met the eligibility criteria, and one (RG) was excluded from the study due to severe and refractory arterial hypotension, requiring intervention with medication not included in the study protocol (Figure 1). Demographic data were similar between the groups (Table 1).

In the MG group, 19 patients had no comorbidities (ASA I) after bariatric surgery and one had arterial hypertension (ASA II). In the RG group, no patient had comorbidities (ASA I).

The comparison of the variation in HR and MAP before and after orotracheal intubation and before and after the surgical incision was performed using Student's *t*-test for independent samples since the assumptions of normality and homogeneity of variances were satisfied. Descriptive statistics and the results obtained by the tests are seen in Table 2.

In Table 2, the variation in mean arterial pressure at intubation was negative for both the groups, indicating that this mean was higher before intubation. Post- and preintubation heart rate variation presents the greatest differences between the groups, the only difference that suggests significance. The others show subtle differences. The *t*-test was used to confirm whether they are significant. For the data MAP variation with orotracheal intubation, MAP variation with surgical incision, HR variation with orotracheal intubation and HR variation with surgical incision, Levene's tests for equality of variances, and *t*-test for equality of means showing no significant differences between the groups for any of the variations studied (Table 3).

**Figure 2:**
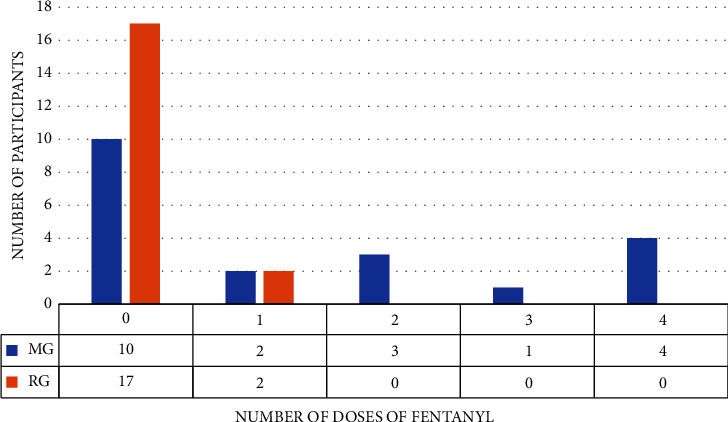
Comparison of fentanyl consumption between the groups. MG, magnesium sulfate group; RG, remifentanil group.

In the MG 50% of the participants did not need intraoperative analgesic supplement, and 10% of them received only 1 *μ*g·kg^−1^ of fentanyl during the entire procedure. As there was no normality in the data collected, the Mann–Whitney test was used and showed that fentanyl consumption in the MG group was significantly higher (*p* = 0.021; Figure 2).

Postoperative opioid consumption was not normally distributed in the groups. The Mann–Whitney test showed no statistical difference between the groups (*p* = 0.061).

A comparison of postoperative pain scores was performed at rest and on movement at the following times: upon awakening and 6 hours after awakening (at rest), and on the 3 postoperative days in the morning and in the afternoon (at rest and at movement). Data were not normally distributed and were analyzed by a nonparametric ANOVA test for repeated measures, and there was no statistical difference between the groups (*p* = 0.933).

A comparison of cisatracurium consumption was performed using Student's *t*-test, which showed statistical similarity between the groups (*p* = 0.809).

**Figure 3:**
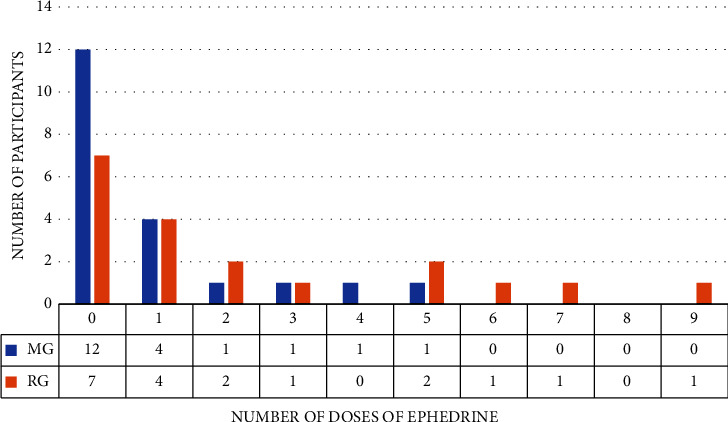
Comparison of ephedrine consumption between the groups. MG, magnesium sulfate group; RG, remifentanil group.

Forty-one percent of RG participants did not need to receive a vasoconstrictor against 60% of the MG participants (Figure 3). The data were also not normal, and the Mann–Whitney test showed that there was no significant difference in the number of doses between the groups (*p* = 0.107).

A comparison of propofol consumption and hypnosis latency between the groups was also performed using the nonparametric Mann–Whitney test due to the lack of normality of the data. The MG group had a significantly higher consumption of propofol (*p* < 0.001). The latency to hypnosis showed no statistical difference between the groups (*p* = 0.569).  Table 4 shows the comparison between the groups.

## 4. Discussion

Magnesium sulfate in adequate doses can replace or reduce the opioid in general anesthesia for post-bariatric dermolipectomy but at the expense of higher propofol consumption.

The use of an intraoperative analgesia index to guide analgesic administration has been advocated by some authors [22–26] and may serve as a criterion for choosing agents and doses. As analgesia is the main desired outcome, we could compare the undesired effects once the desired analgesia is achieved. In this trial, the proposal was to observe the analgesic capacity of the MS for eventual replacements of opioids or reduction of their doses and, consequently, their adverse effects. The desired target-outcome was to maintain a PSI value between 20 and 50, administering remifentanil in one group and MS in the other, with preestablished doses of each agent. Fentanyl at a dose of 1 *μ*g·kg^−1^ bolus as rescue analgesia was used when the PSI was above 50.

The primary outcome was the change in hemodynamic parameters in participants of both the groups before and after orotracheal intubation and before and after surgical incision while maintaining the PSI in the target range. It was similar between the groups. This confirms the potential of MS, in adequate doses, to satisfactorily cover the moments of greatest nociceptive stimulus during the procedure. These data confirm the findings of Silva Filho et al. [21] in a similar study, but with analgesia guided only by hemodynamic parameters. In addition, the data presented a normal distribution, showing that the sample was adequate for the use of the parametric test.

In the MG group, 50% of the patients did not need to receive an analgesic supplement, and 10% received only 1 *μ*g·kg^−1^, in procedures that lasted 156.45 (±22.36) minutes, and even in the RG group, two patients needed an analgesic supplement to maintain the PSI between 20 and 50. This is an essential reduction in intraoperative opioid consumption, which can be converted into an advantage for patients at greater risk of presenting adverse effects to opioids or lesser tolerance to them.

Morphine consumption and postoperative pain scores at rest and in motion assessed until the third postoperative day did not show any statistical difference between the groups. Outliers were observed in both the groups, and one patient in the RG group had postoperative hyperalgesia, which did not improve with bolus morphine repeated three times and was corrected with patient-controlled analgesia until the next day.

The consumption of neuromuscular blockers was similar between the groups, which seems paradoxical, given the potentiation that MS produces on the effect of this class of drugs [28].

To maintain the PSI within the target range in the present study, there was a greater need to use ephedrine when compared to the study by Silva Filho et al. [21], using only hemodynamic parameters as a guide for analgesia. In the previous trial, the RG group consumed more vasopressors (*p* < 0.001), while in the present study, there was no statistical difference between the groups (*p* = 0.107). However, one participant in the RG group had bradycardia and refractory arterial hypotension, which was corrected after the association of atropine and metaraminol. This participant was excluded because metaraminol was not a part of the study protocol. The difference in hemodynamic behavior between the trial and the present one was probably due to the higher consumption of fentanyl in the present study (*p* = 0.021, in the comparison between the groups) when compared to the previous study (*p* = 0.004). Further studies are needed to assess whether this difference is significant in the emergence or importance of unfavorable outcomes.

The latency for propofol hypnosis was similar between the groups. In contrast, the MG group had a higher consumption during anesthesia, which follows a previous study [21], and is probably due to the ability of remifentanil to potentiate the hypnotic effect of this drug. This interference only appears after anesthetic induction, when remifentanil begins to exert its action, potentiating propofol-induced hypnosis.

The objective of this trial was to seek confirmation and safety in the use of MS in relation to its ability to occupy or share a space with opioids in perioperative analgesia, reducing the adverse effects of this class of drugs while preserving physiological targets, such as the autonomic stability offered by opioids [29], but avoiding adverse effects, such as hyperalgesia [30–34] or delay in the return of gastrointestinal function [35–37].

Analgesia monitoring by the PSI is an algorithm already valid for use in general anesthesia [22–26].

Despite demonstrating that the association of magnesium sulfate can reduce and, in many cases, replace the use of opioids, this trial presented a limitation in the sample size, which did not allow the analysis through parametric tests of outcomes such as intraoperative and postoperative consumption of opioids, postoperative analgesia, ephedrine consumption, and hypnosis latency. However, these are data related to secondary outcomes. Thus, the trial was not powered for them.

We conclude that using magnesium sulfate in intravenous general anesthesia for post-bariatric dermolipectomy is related to a significant reduction in opioid consumption without compromising hemodynamic stability. Overall, PSI monitoring was helpful in driving the analgesic strategy. The use of magnesium sulfate proved to be an important adjunct in the scenario presented, allowing the use of opioids to be avoided in certain cases. We found no statistical differences in the consumption of neuromuscular blocker and vasoconstrictor. Substituting opioids for magnesium sulfate leads to an increase in propofol consumption in the scenario presented. Studies with a larger sample are needed to corroborate the results presented and evaluate other potential advantages in reducing opioid consumption.

All resources for conducting the study were shared between authors and institution.

## Figures and Tables

**Table 1:** Descriptive statistics for demographic variables.

			*n*	Minimum	Maximum	Mean	Standard deviation
Group	MG	Age (years)	20	26.00	54.00	40.90	7.05
		Weight (kg)	20	53.00	85.00	68.65	9.25
		Height (m)	20	1.47	1.80	1.64	0.08
		Time (min)	20	119.00	195.00	156.45	22.36
	RG	Age (years)	19	30.00	56.00	39.16	6.87
		Weight (kg)	19	55.00	93.00	74.11	10.45
		Height (m)	19	1.52	1.76	1.64	0.07
		Time (min)	19	140.00	199.00	156.79	15.45

MS, magnesium sulfate group; RG, remifentanil group; *n*, number of participants.

**Table 2:** Descriptive statistics for heart rate and mean blood pressure by the groups.

	Group	*N*	Mean of the variation	Standard deviation	Mean standard error
MAP variation with OTI	MG	19	−1.43	17.56	4.03
	RG	19	−3.96	17.02	3.90

MAP variation with incision	MG	20	1.89	7.38	1.65
	RG	19	1.55	10.89	2.50

HR variation with OTI	MG	20	9.24	15.14	3.39
	RG	19	1.49	9.31	2.14

HR variation with incision	MG	20	1.89	6.42	1.44
	RG	19	1.02	5.79	1.33

MG, magnesium sulfate group; RG, remifentanil group; MAP, mean arterial pressure; HR, heart rate; OTI, orotracheal intubation; *n*, number of participants.

**Table 3:** Student's test for difference in MAP, mean arterial pressure; HR, heart rate; OTI, orotracheal intubation.

		Levene test for equality of variances				*t* test for equality of means		
	Equal variances assumed	Z	Sig.	*t*	d*f*	Sig (2 ends)	Mean difference	Standard error of difference

MAP variation with OTI	Yes	0.400	0.531	0.451	36	0.665	2.52925	5.61097
	No			0.451	35.966	0.665	2.52925	5.61097

MAP variation with incision	Yes	1.750	0.194	0.113	37	0.910	0.33606	2.96516
	No			0.112	31.469	0.911	0.33606	2.99421

HR variation with OTI	Yes	2.738	0.106	1.912	37	0.064	7.74740	4.05114
	No			1.935	31.805	0.62	7.74740	4.00325

HR variation with incision	Yes	0.795	0.378	0.447	37	0.657	0.87781	1.96210
	No			0.449	36.904	0.656	0.87781	1.95677

**Table 4:** Comparison of latency to hypnosis and consumption of propofol between the groups.

	Latency to hypnosis (min)		Consumption (*μ*g·kg^−1^·min^−1^)	
	MG	RG	MG	RG
Minimum	1	1	67	53
Maximum	5	12	221.6	175
Median	3	2	148	90
*Q*1	1.5	1	115	85
*Q*3	4	3	183	106.5

MG, magnesium sulfate group; RG, remifentanil group. *Q*1, first quartile; *Q*3, third quartile.

## Data Availability

The datasets generated and analyzed during the present study are available from the corresponding author on reasonable request.
